# Early outcomes of hybrid coronary revascularization in multivessel coronary artery disease with low ejection fraction

**DOI:** 10.3389/fcvm.2025.1641223

**Published:** 2025-10-31

**Authors:** Göksel Guz, Mustafa Kemal Avşar, Barış Kırat, İbrahim Özgür Önsel, Deniz Yorgancılar, Sabahattin Ateşeal

**Affiliations:** ^1^ Department of Cardiology, Medicana International Istanbul Hospital, Istanbul, Türkiye; ^2^ Department of Cardiovascular Surgery, Faculty of Medicine, Çukurova University, Adana, Türkiye; ^3^ Department of Anesthesiology, Medicana International Istanbul Hospital, Istanbul, Türkiye; ^4^ Department of Thoracic Surgery, Medicana International Istanbul Hospital, Istanbul, Türkiye; ^5^ Department of Cardiology, Medicana International Istanbul Hospital, Istanbul, Türkiye

**Keywords:** hybrid coronary revascularization, multivessel coronary artery disease, low ejection fraction, off-pump CABG, LIMA grafting, PCI

## Abstract

**Background:**

Hybrid coronary revascularization (HCR), combining left internal mammary artery (LIMA) grafting to the left anterior descending artery (LAD) with percutaneous coronary intervention (PCI) for non-LAD lesions, has emerged as a potential strategy in patients with multivessel coronary artery disease (CAD) and severely reduced left ventricular ejection fraction (EF). However, data regarding its outcomes in this high-risk group remain limited.

**Objectives:**

To evaluate the early-term safety, feasibility, and clinical outcomes of HCR in patients with multivessel CAD and left ventricular dysfunction (EF 20%–35%).

**Methods:**

This retrospective, single-center study included 50 consecutive patients with multivessel CAD and EF between 20% and 35% who underwent HCR between January 2022 and December 2024. HCR was performed with PCI for non-LAD lesions, followed by off-pump LIMA-to-LAD grafting. The primary endpoints were 30-day all-cause mortality and major adverse cardiac events (MACE). Secondary endpoints included hospital length of stay, perioperative complications, and 12-month outcomes.

**Results:**

The mean patient age was 65.4 ± 8.2 years, with 58% being male. Technical success was achieved in 96% of cases. The 30-day mortality rate was 2%, and the 30-day MACE rate was 8%, including myocardial infarction (4%), repeat revascularization (2%), and ischemic stroke (2%). New-onset atrial fibrillation occurred in 18% of patients, transient renal dysfunction in 10%, and prolonged ventilation (>24 h) in 4%. The mean hospital stay was 7.3 ± 2.1 days. At 12 months, the MACE-free survival rate was 88%, with a LIMA-LAD graft patency of 100% and saphenous vein graft patency of 92%. There was a modest improvement in EF from 28.6 ± 4.1% to 30.1 ± 4.5% (*p* = 0.12). Follow-up coronary angiography was performed in 48 patients (96%) at one year.

**Conclusions:**

HCR appears to be a feasible and relatively safe revascularization strategy for patients with multivessel CAD and severely reduced EF, offering acceptable early mortality and MACE rates. The excellent graft patency and low perioperative complication rates suggest that HCR may be a valuable alternative in this high-risk population, although larger multicenter trials are needed to confirm these findings.

## 1 Introduction

Coronary artery disease (CAD) remains a leading cause of cardiovascular mortality worldwide (1). In patients with a left ventricular ejection fraction (EF) of less than 35%, the disease exhibits a more aggressive course, and revascularization strategies become increasingly complex (2, 3). In this high-risk population, conventional coronary artery bypass grafting (CABG) is associated with elevated perioperative complications and morbidity (4). Conversely, percutaneous coronary intervention (PCI) faces limitations due to anatomical complexity and challenges in achieving complete revascularization (5).

In recent years, hybrid coronary revascularization (HCR) has emerged as an alternative strategy, combining the advantages of both approaches. HCR integrates surgical revascularization of the left anterior descending artery (LAD) using a left internal mammary artery (LIMA) graft with PCI using drug-eluting stents for non-LAD lesions (6, 7). This approach leverages the long-term patency benefits of surgical intervention and the minimally invasive nature of PCI, facilitating a faster recovery (8).

However, data on the efficacy and safety of HCR in patients with low EF (<35%) remain limited. Existing studies have often excluded this patient group or have not specifically reported outcomes for this cohort. Off-pump surgical revascularization combined with PCI in low-EF patients may offer potential advantages by mitigating the adverse effects of cardiopulmonary bypass (9, 10).

In this context, our study includes 50 high-risk patients with EF between 20% and 35% and multivessel CAD. These patients underwent HCR, involving PCI for non-LAD lesions followed by off-pump LIMA-to-LAD grafting. The aim of this study is to evaluate the early-term safety, feasibility, and clinical efficacy of HCR in this specific patient population.

## 2 Material and methods

### 2.1 Study design

This retrospective, single-center cohort study evaluated 50 consecutive patients who underwent hybrid coronary revascularization (HCR) at the Departments of Cardiology and Cardiovascular Surgery at [Medicana International Istanbul Hospital] between January 2022 and December 2024. The study aimed to assess the early-term safety, feasibility, and clinical efficacy of HCR in high-risk patients with low ejection fraction (EF 20%–35%) and multivessel coronary artery disease (CAD). The study protocol was approved by the local institutional ethics committee (2025/14), and all procedures were conducted in accordance with the Declaration of Helsinki. Written informed consent was obtained from all patients.

### 2.2 Patient population

The study included 50 patients with a left ventricular EF of 20%–35%, as measured by transthoracic echocardiography, and angiographically confirmed multivessel CAD. Multivessel CAD was defined as ≥70% critical stenosis in at least one non-left anterior descending artery (LAD) vessel (circumflex artery [Cx], obtuse marginal [OM1-OM2], intermediary [IM], or right coronary artery [RCA]) in addition to an LAD lesion (SYNTAX score ≥22). Inclusion criteria were:

• Diagnosis of stable coronary artery disease.
• ≥70% stenosis in the LAD suitable for surgical revascularization.
• Non-LAD lesions amenable to percutaneous coronary intervention (PCI).
• Suitability for HCR, as determined by a heart team evaluation involving cardiologists and cardiovascular surgeons.

Exclusion criteria included:

• Acute myocardial infarction (STEMI or NSTEMI) within 30 days prior to the study.
• Cardiogenic shock.
• Severe aortic stenosis or regurgitation.
• Prior coronary artery bypass grafting (redo CABG).
• Active infection.
• Terminal malignancy.

In patients where non-LAD lesions were surgically grafted, the decision was made preoperatively by the Heart Team due to complex anatomical features that rendered PCI infeasible.

### 2.3 Procedure

HCR was performed as a two-stage approach:

1. **Percutaneous Coronary Intervention (PCI) Stage**: Non-LAD vessels (Cx, OM1-OM2, IM, RCA) were revascularized using drug-eluting stents (e.g., zotarolimus- or everolimus-eluting stents). PCI procedures were performed via standard femoral or radial artery access, guided by fluoroscopy and in accordance with modern PCI protocols. Intravascular ultrasound (IVUS) or optical coherence tomography (OCT) was used for lesion assessment when deemed necessary. Complete revascularization was targeted.
2. **Surgical Stage (OPCAB)**: Within the same session or the following day after PCI, off-pump coronary artery bypass grafting was performed using a LIMA graft to the LAD. In cases where surgery was scheduled on the same day, PCI was completed in the early morning and clopidogrel was not loaded. In those undergoing surgery the following day, clopidogrel was administered immediately after PCI and held for 12–24 h before surgery to minimize bleeding risk. This protocol ensured adequate antiplatelet coverage while balancing perioperative bleeding concerns. Saphenous vein grafts were used for Diagonal 1 (D1) and RCA lesions. All surgical procedures were conducted via median sternotomy without cardiopulmonary bypass (CPB). Anastomoses were performed using side-biting clamps and stabilizer devices with 7-0 or 8-0 polypropylene sutures. Distal anastomoses were performed using a standard off-pump technique. Intracoronary shunts were routinely used to preserve distal myocardial perfusion during anastomosis. Transit-time flow measurement (TTFM) was used intraoperatively in all patients to evaluate graft flow and confirm anastomotic patency.

### 2.4 Antiplatelet and anticoagulant management

Dual antiplatelet therapy (aspirin 100 mg/day and clopidogrel 75 mg/day) was initiated immediately after PCI. Clopidogrel was withheld 12–24 h before surgery to minimize bleeding risk, while aspirin was continued perioperatively. Both agents were resumed within 24 h post-surgery and continued for at least 12 months. Standard anti-ischemic therapy (beta-blockers, ACE inhibitors, statins) was administered pre- and post-procedure.

### 2.5 Outcome measures

#### 2.5.1 Primary outcome measures

• 30-day all-cause mortality.
• 30-day major adverse cardiac events (MACE): myocardial infarction, stroke, or need for repeat revascularization.

#### 2.5.2 Secondary outcome measures

• Hospital length of stay.
• Need for postoperative blood transfusion.
• Intraoperative and postoperative complications (acute kidney injury, atrial fibrillation, ventilation duration).
• Change in EF during follow-up (measured by transthoracic echocardiography preoperatively and at 6 months postoperatively).

### 2.6 Data collection and assessment

Demographic characteristics (age, sex, diabetes, hypertension), clinical features (SYNTAX score, EF), procedural details (number of stents, surgical duration), and early-term outcomes were recorded. Echocardiographic assessments were performed pre-procedure and at 6 months post-procedure. Technical success was defined as LIMA-LAD graft patency and <20% residual stenosis in non-LAD lesions.

### 2.7 Statistical analysis

Data were analyzed using SPSS Statistics (IBM Corp., Armonk, NY, USA) version 26.0. Continuous variables were expressed as mean ± standard deviation (SD) or median (interquartile range), with comparisons performed using the Student's *t*-test for normally distributed data and the Mann–Whitney *U* test for non-normally distributed data. Categorical variables were presented as numbers and percentages (%) and compared using the *χ*^2^ test or Fisher's exact test. Event-free survival rates were analyzed using the Kaplan–Meier method. A *p*-value <0.05 was considered statistically significant.

### 2.8 Ethical approval

The study protocol was approved by the local institutional ethics committee (2025/14). All procedures were conducted in accordance with the Declaration of Helsinki.

## 3 Results

### 3.1 Patient demographics and clinical characteristics

The study included 50 patients with a mean age of 65.4 ± 8.2 years, of whom 58% (*n* = 29) were male. The mean left ventricular ejection fraction (EF) was 28.6 ± 4.1%. Diabetes mellitus was present in 42% (*n* = 21), hypertension in 68% (*n* = 34), hyperlipidemia in 64% (*n* = 32), smoking history in 54% (*n* = 27), and prior myocardial infarction in 36% (*n* = 18) of patients. The mean SYNTAX score was 29.5 ± 6.8. Patient demographic and clinical characteristics are summarized in Table 1.

**Table 1:** Patient demographic and clinical characteristics.

Characteristic	Value
Age (years)	65.4 ± 8.2
Male sex, *n* (%)	29 (58%)
Body mass index (kg/m^2^)	27.3 ± 3.4
Diabetes mellitus, *n* (%)	21 (42%)
Hypertension *n* (%)	34 (68%)
Hyperlipidemia *n* (%)	32 (64%)
Smoking history *n* (%)	27 (54%)
Prior myocardial infarction *n* (%)	36 (*n* = 18)
EF (%)	28.6 ± 4.1
SYNTAX score (mean)	29.5 ± 6.8

**Table 2:** Early and follow-up outcomes.

Outcome	Outcome
30-day mortality (%)	2% (*n* = 1)
30-day MACE (%)	8% (*n* = 4)
Hospital length of stay (days)	7.3 ± 2.1
Postoperative blood transfusion (%)	14% (*n* = 7)
12-Mo MACE-free survival (%)	88% (95% CI: 78–95)

### 3.2 Procedural characteristics

Hybrid coronary revascularization (HCR) was successfully completed as planned in all patients. During the percutaneous coronary intervention (PCI) stage, a total of 78 drug-eluting stents (mean 1.6 ± 0.7 stents/patient) were implanted in non-left anterior descending (LAD) vessels, including the circumflex artery (Cx), obtuse marginal branches (OM1-OM2), and intermediary artery (IM). Intravascular ultrasound (IVUS) was utilized in 24% (*n* = 12) and optical coherence tomography (OCT) in 16% (*n* = 8) of patients. The technical success rate for PCI was 98% (*n* = 49). In the surgical stage, all patients underwent left internal mammary artery (LIMA) anastomosis to the LAD; 10% (*n* = 5) received additional saphenous vein grafts to Diagonal 1 (D1) or right coronary artery (RCA) (mean graft count: 1.3 ± 0.5/patient). All surgical procedures were performed off-pump via median sternotomy, with no requirement for cardiopulmonary bypass (CPB), emergency conversion, or inotropic support. The mean surgical duration was 138 ± 25 min, and the mean hospital length of stay was 7.3 ± 2.1 days. The overall technical success rate, defined as LIMA-LAD graft patency and <20% residual stenosis in non-LAD lesions, was 96% (*n* = 48).

### 3.3 Primary outcome measures

The 30-day all-cause mortality rate was 2% (*n* = 1), with the death attributed to sepsis-related multiorgan dysfunction. The 30-day major adverse cardiac event (MACE) rate was 8% (*n* = 4), comprising:

• Myocardial infarction: 4% (*n* = 2),
• Repeat revascularization: 2% (*n* = 1, via PCI),
• Ischemic stroke: 2% (*n* = 1).

### 3.4 Secondary outcome measures

Postoperative blood transfusion was required in 14% (*n* = 7) of patients. Intraoperative and postoperative complications included new-onset atrial fibrillation (18%, *n* = 9), transient renal dysfunction (10%, *n* = 5), and prolonged ventilation (>24 h, 4%, *n* = 2). The mean ventilation duration was 8.1 ± 3.0 h. EF, measured by transthoracic echocardiography, increased from 28.6 ± 4.1% preoperatively to 30.1 ± 4.5% at 6 months postoperatively, but this change was not statistically significant (*p* = 0.12). Hospital length of stay was significantly longer in patients with complications (8.5 ± 2.4 days) compared to those without (6.8 ± 1.9 days, *p* = 0.008). Early and follow-up outcomes are summarized in Table 2.

### 3.5 Follow-up outcomes

**Figure 1:**
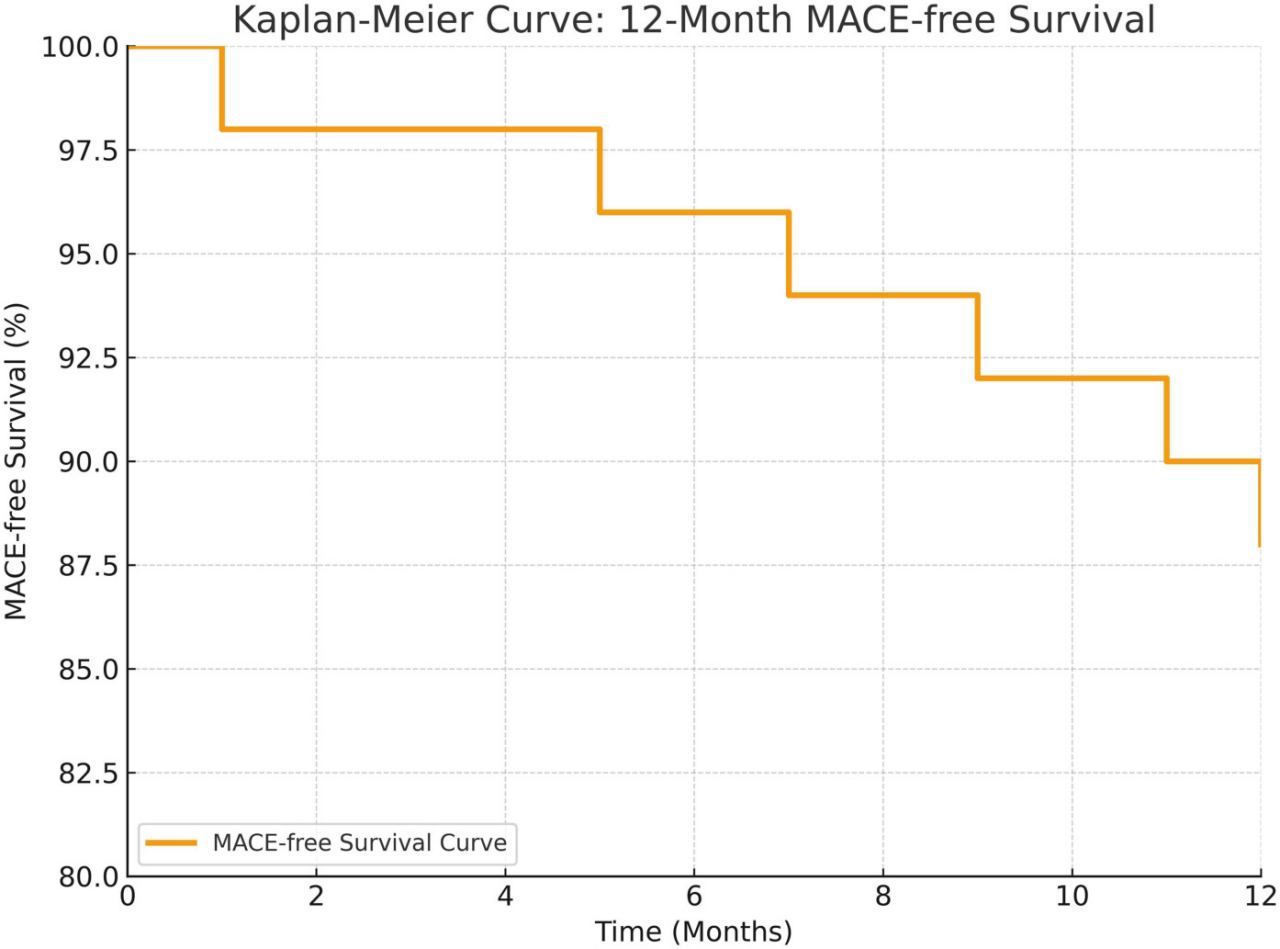
Kaplan–Meier curve showing 12-month MACE-free survival. Overall MACE-free survival at 12 months was 88% (95% CI: 78–95).

**Figure 2:**
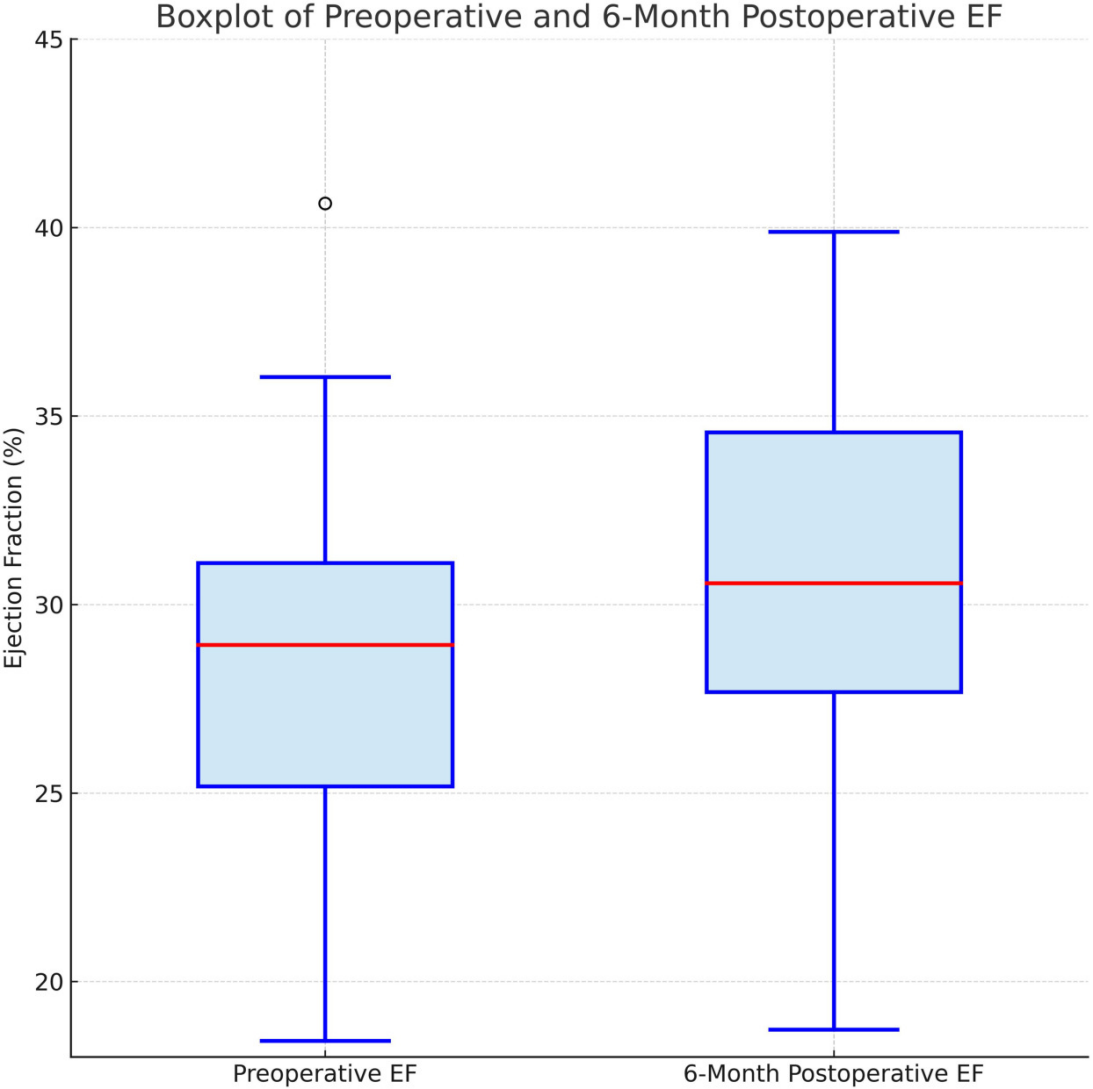
Boxplot illustrating preoperative and 6-month postoperative ejection fraction (EF) values. No statistically significant difference was observed between the two time points (*p* = 0.12).

All patients were followed up at 1, 3, 6, and 12 months, and annually thereafter. At one year, follow-up coronary angiography was successfully performed in 48 patients (96%) to assess graft patency and disease progression. At 12 months, the all-cause mortality rate was 4% (*n* = 2), with the second death attributed to heart failure. The 12-month MACE rate was 12% (*n* = 6), including myocardial infarction (*n* = 2), stroke (*n* = 1), and repeat revascularization (*n* = 3). In 20 patients who underwent control coronary angiography at 6 months, LIMA-LAD graft patency was 100%, and saphenous vein graft patency was 92%. Kaplan–Meier analysis, as depicted in Figure 1, estimated a 12-month MACE-free survival rate of 88% (95% CI: 78–95). Diabetes was significantly associated with MACE (*p* = 0.03), but no significant associations were found with age, sex, or SYNTAX score (*p* > 0.05). Comparison of preoperative and 6-month postoperative ejection fraction (EF) revealed no statistically significant difference (preoperative EF: 28.6 ± 4.1%, postoperative EF: 30.1 ± 4.5%; *p* = 0.12). These findings are illustrated in Figure 2.

## 3 Discussion

The optimal revascularization strategy for patients with multivessel coronary artery disease (CAD) and severe left ventricular dysfunction remains a subject of ongoing debate. The landmark STICH trial demonstrated that coronary artery bypass grafting (CABG) improves long-term survival in patients with ischemic cardiomyopathy, establishing it as a cornerstone therapy for this population (2). However, contemporary evidence from the REVIVED-BCIS2 trial indicates that percutaneous coronary intervention (PCI) in patients with severely reduced left ventricular ejection fraction (EF) does not reduce mortality or heart failure hospitalizations compared to optimal medical therapy alone (11). These findings highlight the limited efficacy of isolated PCI in this high-risk cohort while supporting the potential benefits of complete surgical revascularization. In this context, hybrid coronary revascularization (HCR)—combining left internal mammary artery (LIMA) grafting to the left anterior descending artery (LAD) with PCI for non-LAD lesions—has emerged as a compelling alternative, particularly for patients with elevated surgical risk. Recent comprehensive reviews have summarized the indications, techniques, and clinical outcomes of hybrid coronary revascularization (HCR), highlighting its potential benefits in selected high-risk populations (12).

Recent meta-analyses have demonstrated that HCR yields midterm and long-term clinical outcomes comparable to conventional CABG. For instance, Nagraj et al.'s 2023 meta-analysis, encompassing 14 studies and 4226 patients, found no significant difference in 5-year mortality or major adverse cardiac and cerebrovascular events (MACCE) between HCR and CABG, suggesting equivalence in appropriately selected patients (13). Similarly, Liang et al.'s 2022 meta-analysis reported that perioperative, short-term, and midterm MACCE rates in the HCR group were comparable to those in the CABG group, although higher reintervention rates were noted in long-term follow-up (14). Our study, involving 50 patients with EF ranging from 20% to 35%, aligns with these findings, reporting a 30-day all-cause mortality rate of 2% (*n* = 1, due to sepsis-related multiorgan dysfunction), a 30-day MACE rate of 8% (myocardial infarction 4%, repeat revascularization 2%, ischemic stroke 2%), and a 12-month MACE-free survival rate of 88%. These results are consistent with the literature, where 30-day mortality in high-risk HCR cohorts ranges from 0% to 2%, and 1-year MACE-free survival approximates 88%–89% (e.g., HREVS trial, 2023) (10). As summarized in Table 3, our results are consistent with previous HCR studies reporting low perioperative mortality and acceptable 1-year outcomes, particularly in high-risk cohorts.

**Table 3:** Summary of reported outcomes in hybrid coronary revascularization studies.

Study	Design	Patients (*n*)	EF (%)	30-day mortality	1-year MACE-free survival	Comments
Velazquez et al. (2)	RCT	1,212	<35%	4.0%	N/A	STICH trial, CABG vs. medical therapy
Harskamp et al. (6)	Meta-analysis	3,439	Mixed	1.2%	∼89%	HCR vs. CABG
Li et al. (15)	Matched retrospective	302	∼30%	1.3%	90%	HCR vs. OPCABG
Newman et al. (16)	Single-center retrospective	395	Not stated	0.25%	∼94%	10-year outcomes
Dong et al. (20)	Meta-analysis	9 studies	Mixed	∼1.5%	∼88%	HCR vs. OPCABG, short- and mid-term
Our study 2025	Single-center retrospective	50	28.6 ± 4.1%	2%	88%	High-risk patients, LIMA-LAD + PCI

One of the primary advantages of HCR lies in its ability to reduce invasiveness, leading to significant early-term benefits. Li et al.'s study, which matched 151 HCR patients with 151 off-pump CABG patients, reported a markedly lower incidence of new-onset atrial fibrillation in the HCR group (5.3% vs. 15.2%) and a reduced need for blood transfusion (23.8% vs. 53.0%) (15). Additionally, mechanical ventilation duration and hospital length of stay were significantly shorter in the HCR cohort. In our series, the incidence of new-onset atrial fibrillation was 18%, transient renal dysfunction 10%, and prolonged ventilation (>24 h) 4%, with a mean hospital stay of 7.3 ± 2.1 days—longer in patients with complications (8.5 ± 2.4 days vs. 6.8 ± 1.9 days, *p* = 0.008). In the 10-year experience reported by Newman et al., the incidence of new-onset atrial fibrillation was 11%, acute kidney injury 9%, and prolonged ventilation (>24 h) 5%, with a mean hospital stay of 6.2 ± 1.8 days—significantly longer in patients who developed complications (7.8 ± 2.2 days vs. 5.7 ± 1.5 days, *p* < 0.01) (16). These findings support the literature's assertion that mini-thoracotomy and off-pump techniques in HCR mitigate surgical trauma, facilitating faster recovery (17). Moreover, the avoidance of aortic cross-clamping and cardiopulmonary bypass (CPB) in HCR has been hypothesized to reduce stroke risk, a notion partially supported by some studies reporting lower stroke rates in HCR vs. CABG (18). However, meta-analyses, including those by Liang et al., have not consistently demonstrated a statistical difference in perioperative myocardial infarction or stroke rates between the two approaches (14). In our cohort, the absence of early stroke and a low perioperative myocardial infarction rate (4%) corroborate these observations, suggesting HCR's safety even in high-risk patients. Despite these benefits, HCR is not without limitations, particularly regarding long-term outcomes. The partial surgical revascularization strategy inherent in HCR, relying on stenting for non-LAD vessels, may increase the risk of repeat revascularization. Liang et al.'s meta-analysis reported a 3.5-fold higher repeat revascularization rate in the perioperative period (∼30 days), a 3-fold increase at 1 year, and a 2.8-fold increase over 1–5 years in the HCR group compared to CABG (14). Similarly, Nagraj et al. noted a significantly lower repeat intervention rate with CABG (odds ratio ∼1.5 favoring CABG), attributing this to progressive disease or stent restenosis in stented vessels (13). In our study, the 12-month MACE rate of 12% (including 2% repeat revascularization) aligns with this trend, though the small sample size limits definitive conclusions. This underscores the importance of achieving complete revascularization during HCR planning, as incomplete revascularization has been associated with poorer long-term survival (5-year survival 91% with complete vs. 64% with incomplete revascularization, per a 2022 analysis) (10).

Comparisons with multivessel PCI further highlight HCR's potential advantages. Van den Eynde et al.'s 2021 meta-analysis demonstrated that HCR patients had a lower risk of myocardial infarction (odds ratio ∼0.40, *p* = 0.01) and target vessel revascularization (odds ratio ∼0.49, *p* < 0.001) compared to PCI alone, though long-term MACCE differences did not reach statistical significance (19). In our cohort, the addition of LIMA-LAD grafting likely contributed to the acceptable 12-month MACE rate, given the complex anatomy precluding complete PCI-based revascularization. Newman et al.'s 2024 study of 395 HCR patients reported a 30-day mortality of 0.25% and a 10-year survival rate of 92%–94%, irrespective of whether MIDCAB or PCI was performed first (16). These outcomes suggest that HCR's flexibility in sequencing and its ability to leverage LIMA's durability offer significant clinical benefits.

Supporting literature consistently highlights HCR's reduction in complications such as deep sternal wound infections and intensive care unit stay, particularly with minimally invasive techniques like robotic LIMA harvesting (12, 17). However, cautionary studies emphasize the methodological heterogeneity of existing evidence, with many being retrospective and subject to selection bias (14). Surgeons often select HCR for patients with aortic calcification or high EuroSCORE, potentially skewing outcomes. Liang et al. reported a lower long-term mortality with HCR (odds ratio 0.35), possibly reflecting this bias (14). The lack of large-scale, multicenter randomized controlled trials (RCTs) remains a critical gap, as noted in the 2023 systematic review, with ongoing trials yet to provide conclusive results (14).

Our study's findings, with a 30-day mortality of 2%, no early strokes, and an 18% atrial fibrillation rate, are consistent with the literature's depiction of HCR's perioperative advantages in high-risk patients. The 12-month MACE rate of 12% and 6-month graft patency rates (LIMA-LAD 100%, SVG 92%) further support HCR's efficacy, though the small sample size and single-center design limit generalizability. The modest EF improvement (28.6 ± 4.1% to 30.1 ± 4.5%, *p* = 0.12) may reflect the short follow-up, a limitation also noted in larger studies (15). Similarly, Dong et al.'s meta-analysis comparing HCR with off-pump CABG reported comparable short- and midterm outcomes, with trends favoring HCR in early morbidity parameters, further supporting its role in high-risk patient populations (20).

Future RCTs are essential to clarify HCR's long-term impact, particularly in addressing repeat revascularization risks through optimized stent technology and complete revascularization strategies. In conclusion, HCR represents a promising alternative for high-risk patients with multivessel CAD and severe LV dysfunction, balancing LIMA's survival benefits with reduced early morbidity, pending further robust evidence.

## 4 Conclusion

Staged hybrid coronary revascularization appears to be a feasible and safe strategy in selected high-risk patients. Our findings support its use in carefully selected cases, but larger multicenter trials are needed to validate these results.

## Data Availability

The datasets generated and analyzed during the current study are not publicly available due to patient privacy and institutional restrictions but are available from the corresponding author on reasonable request with appropriate ethical approval.
